# Supplementary material for: Evaluating the effects of CD8/CD4 on T cell function in terms of TCR–pMHC–coreceptor catch and slip bonds
Source: Front Immunol. 2026 Jun 5;17:1757198. doi: 10.3389/fimmu.2026.1757198 (PMC13279612; doi:10.3389/fimmu.2026.1757198)
Supplement: Supplementary file 1 [file DataSheet1.docx]

Supplementary Material

# Supplementary Figures and Tables

This file includes: Supplementary figures 1 to 10 and Supplementary Tables 1 and 2

##
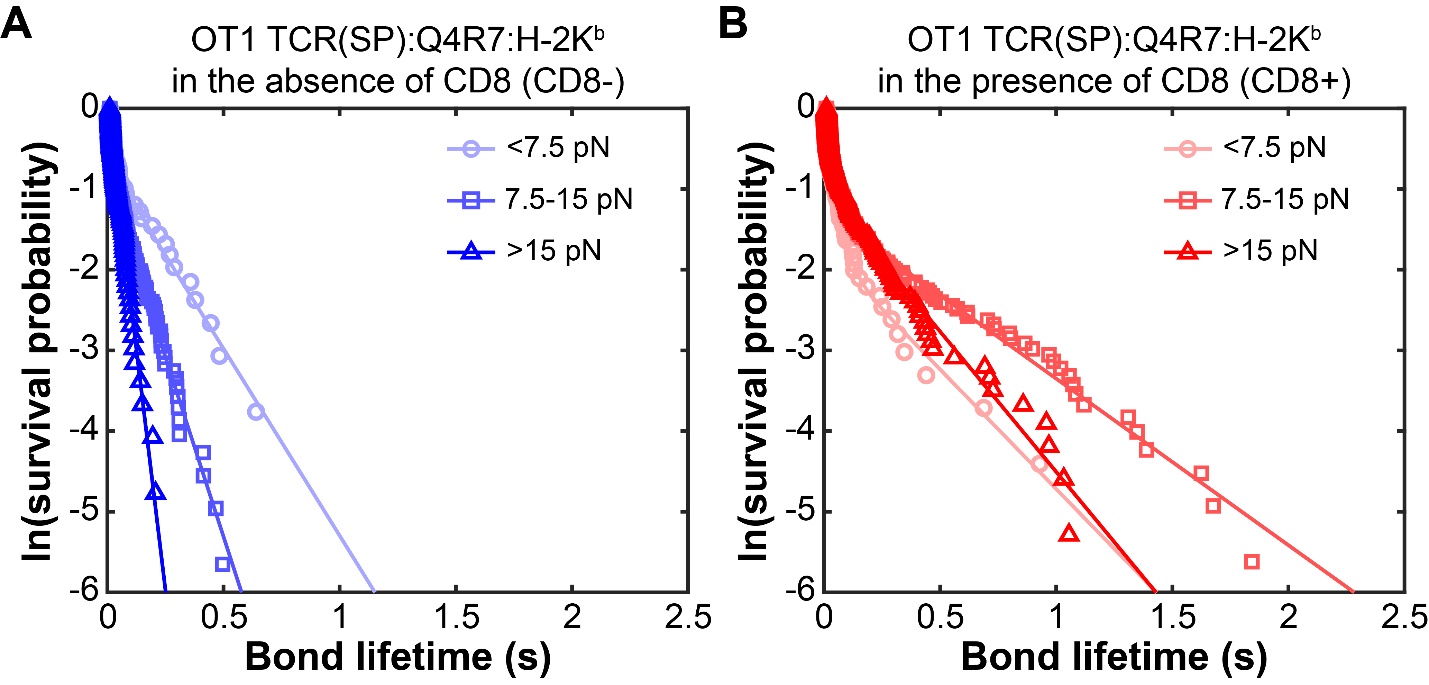


## Supplementary Figure 1. Distributions of lifetimes of bonds of CD8+ OT1 T cell with MT or WT pMHC to prevent or permit CD8 binding at representative forces.

**(A, B)** *Points:* Semi-log survival probability plots of $\mathbf{ln}$(# of events with a lifetime $\boldsymbol{>t}$) *vs* lifetime *t* of single bonds between OT1 SP naïve T cells and the altered peptide Q4R7 presented by H2-K^b^α3A2 (A) or H2-K^b^ (B) at the indicated forces. The curves show pooled ensembles of lifetimes of OT1 TCR–pMHC bonds sorted according to their durations*.* For each force range, the natural log of the number of events with a lifetime ≥ x-axis value was plotted and fitted by a straight line. The catch and slip bonds can be identified from the changes in decay rate with increasing force in these plots because the steeper the decay, the faster the dissociation.


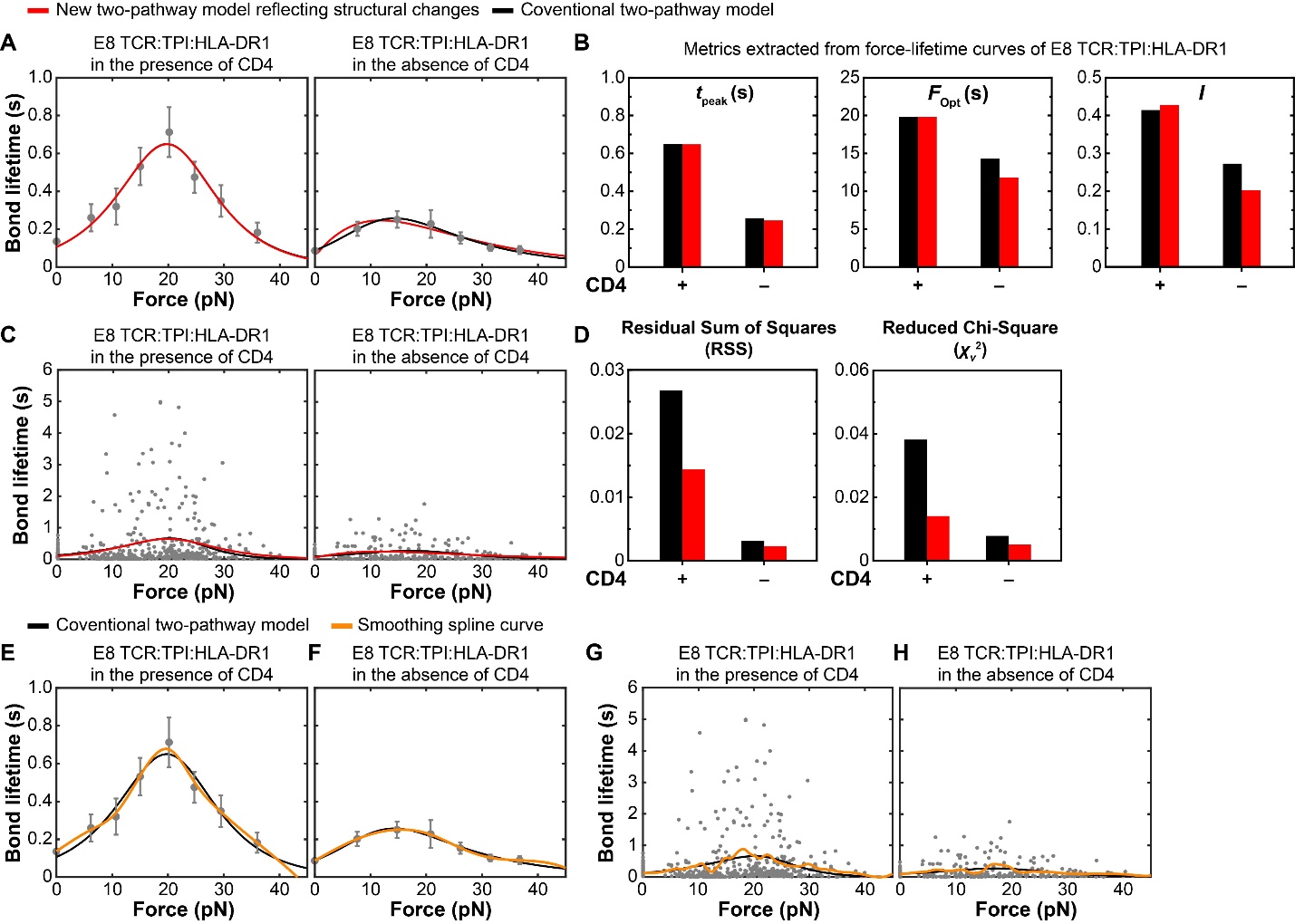


## Supplementary Figure 2. Comparison between the conventional and the structural-based two pathway models.

**(A)** Fitting of the predicted ($1/k(F)$ curves, *black* = conventional two-pathway model, *red* = structural-based two-pathway model) to the measured (points, Mean ± SEM from n > 20 individual measurements per force bin) lifetime *vs* force curves of the TP1 peptide presented by purified HLA-DR1 interacting with purified E8 TCR in the presence (*left*) and absence (*right*) of CD4. The black curve on the left panel is obscured due to overlapping with the red curve. (**B)** Bar graphs of $t_{\mathrm{peak}}$ (1^st^ panel), $F_{\mathrm{opt}}$ (2^nd^ panel), and $I$ (3^rd^ panel) calculated from the fitted curves shown in (A). **(C)** Fitting of the predicted ($1/k(F)$ curves, *black* = conventional two-pathway model, *red* = structural-based two-pathway model) to individual lifetime *vs* force measurements without binning. **(D**) Comparison of residual sum of squares (RSS, *left*) and reduced Chi-square values ($\chi_{\nu}^{2}$, *right*) obtained from the fitting in (C). Data are from our previous publication (1). **(E, F)** Comparison of fitting by the conventional two-pathway model (*black curves*) and by the splining model (*orange curves*) to the same binned data in A. **(G, H)** Comparison of fitting by the conventional two-pathway model (*black curves*) and by the splining model (*orange curves*) to the same un-binned individual lifetime *vs* force measurements in (C).


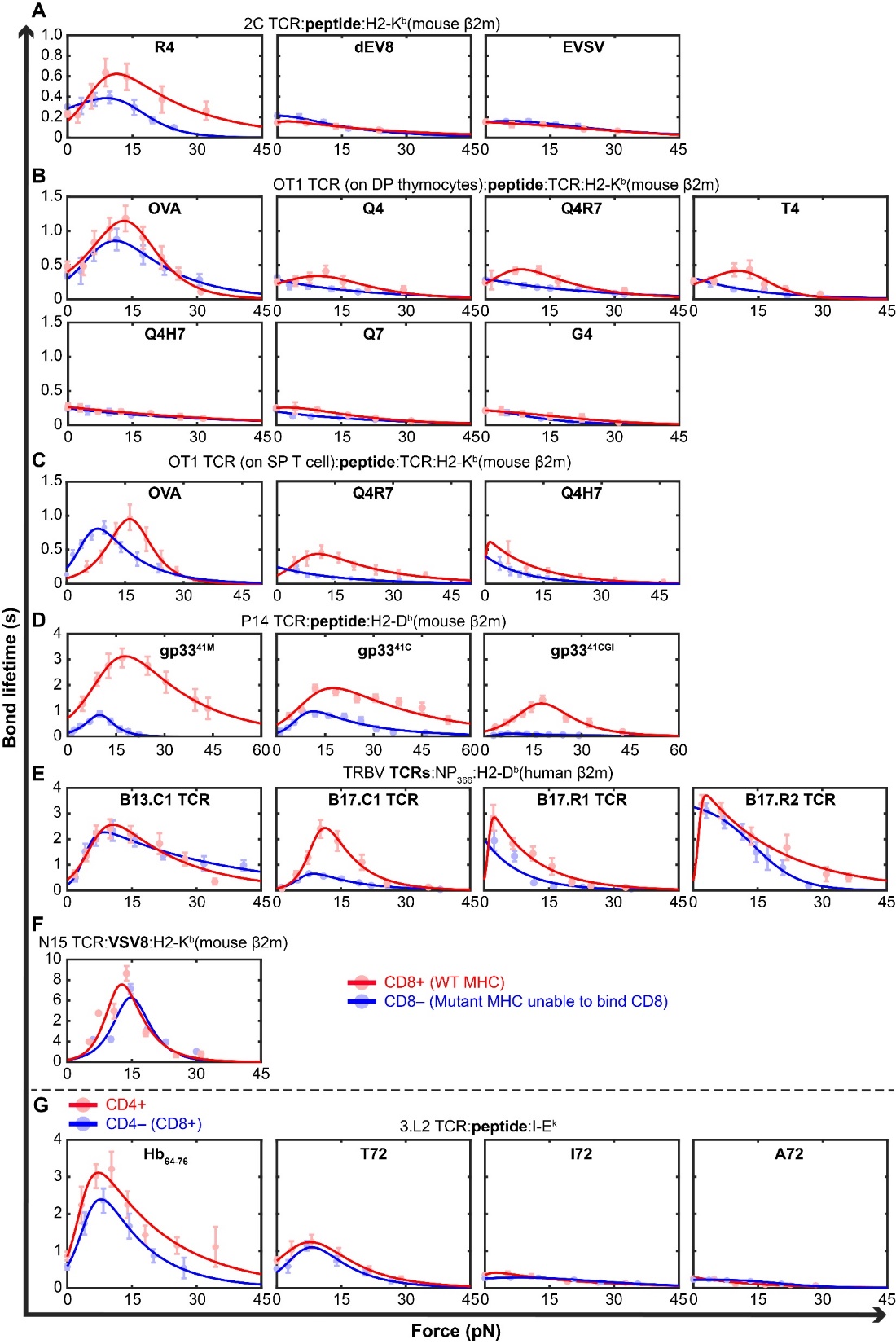


## Supplementary Figure 3. Fitting the conventional two-pathway model to data of lifetime of T cell bonds *vs* force measured in the presence and absence of coreceptor binding.

**(A-G)** Fitting of theoretical $1/k(F)$ curves predicted by the two-pathway model to experimental lifetime (points, Mean ± SEM from at least n > 20 individual measurements per force bin) *vs* force data of 2C **(A)** (2), DP thymocyte OT1 **(B)** (2), SP T cell OT1 **(C, this study and (3))**, P14 **(D)** (4), TRBV **(E)** (5), and N15 **(F)** (6) TCRs interacting with the indicated peptides presented by WT or MT H2-K^b^ or H2-D^b^ **(A-F)**, and 3.L2 TCR expressed on CD4^+^CD8^-^ or CD4^-^CD8^+^ naïve T cells interacting with the indicated peptides presented by I-E^k^ **(G)** (7, 8) to permit (red point and curve) or prevent (blue point and curve) coreceptor from binding. Previously published data references can be found in Supplementary Table 1. Note that the data in main Fig. 1D are replotted here in (B) (the OVA, T4, and G4 panels) for completeness.

##
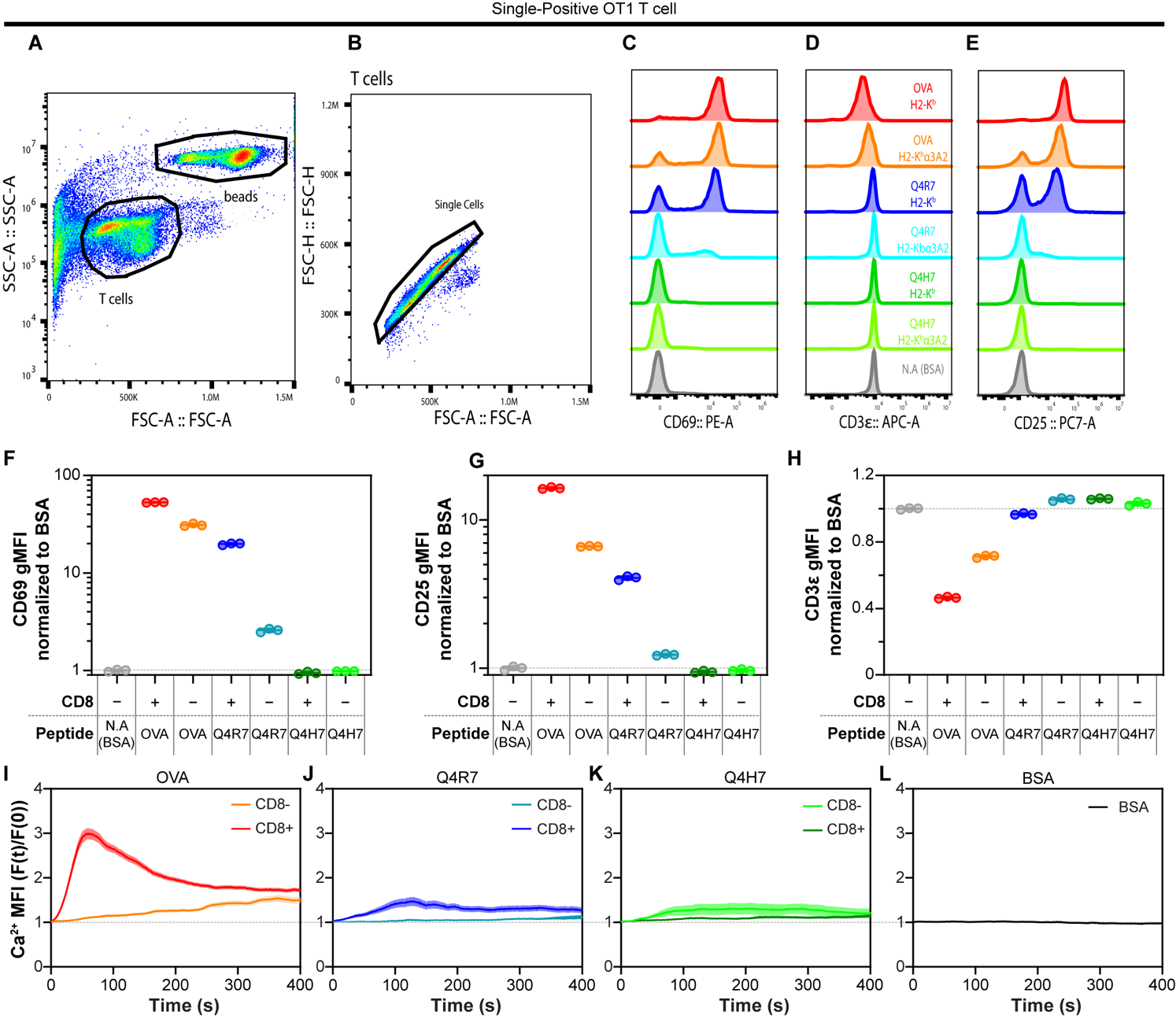


## Supplementary Figure 4. Functional response of SP naïve OT1 T cells to pMHC stimulations.

**(A, B)** Representative flow cytometry plots illustrating the sequential gating strategy used to isolate the target cell population. T cells pre-enriched using a CD8+ T cell purification kit prior to the assay were identified based on morphological properties using forward and side scatter area (FSC-A vs. SSC-A) to exclude debris and beads. Single cells were subsequently gated using FSC-H vs. FSC-A to exclude doublets and aggregates. (**C-E**) Representative histograms depict the expression of activation markers CD25 (C), CD69 (D), and CD3 (E) on the gated Single Cells population co-incubated with 56 pN TGT beads coated by OVA H2-Kb (red), OVA H2-Kb α3A2 (orange), Q4H7 H2-Kb (dark green), Q4H7 H2-Kb α3A2 (light green), Q4R7 H2-Kb (purple), and Q4R7 H2-Kb α3A2 (cyan) peptides. Unstimulated Control histogram is depicted in gray. **(F-H)** Upon 6 hours stimulation by surfaces coated with 20 μg/ml of BSA (negative control) or 20 μg/ml of OVA, Q4R7, or Q4H7 (indicated) presented by H2-K^b^ (CD8+) or H2-K^b^α3A2 (CD8-) at triplicate per condition, the expressions on SP naïve OT1 T cells of CD69 (F), CD25 (G), and CD3 (H) were evaluated by flow cytometry using a fluorescently tagged antibody cocktail (see methods). The geometric mean fluorescence intensity (gMFI) of each experimental sample was normalized by that of BSA. **(I-L)** SP naïve OT1 T cells were loaded with the 5 μM calcium dye (X-Rhod-1) and placed on surfaces coated with 20 μg/ml of OVA (I), Q4R7 (J), or Q4H7 (K) presented by H2-K^b^α3A2 (CD8-) or H2-K^b^ (CD8+), or BSA (L), The changes in intracellular calcium signals were measured by live cell imaging using a fluorescence microscope at 0.5s frames per second over 12 mins. The mean fluorescence intensity (MFI) of each cell in each timeframe was normalized by that of its landing frame (set at time = 0 for that cell). Data in (I-L) are presented as mean ± SEM of 59-288 cells.


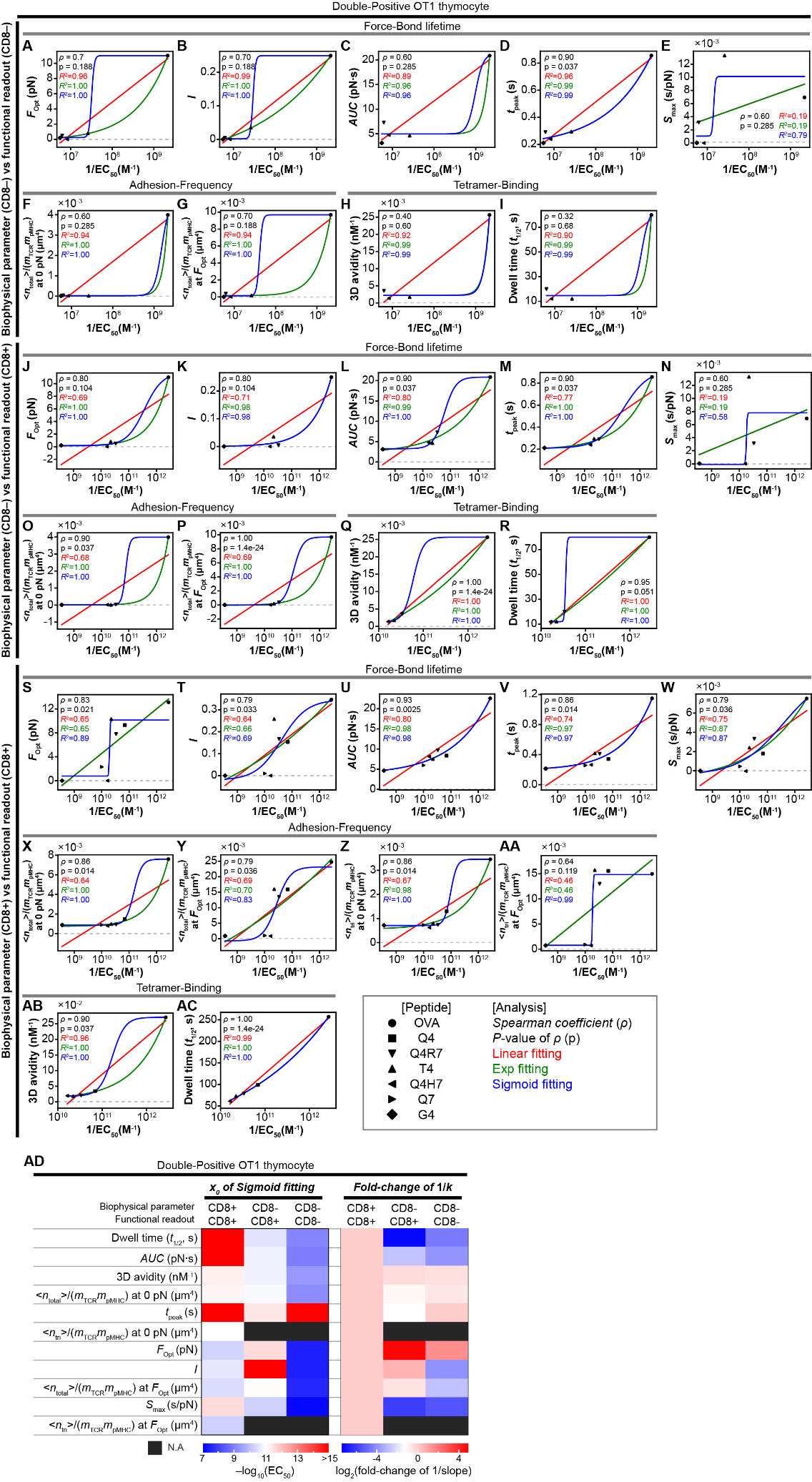


## **Supplementary Figure 5. Correlative analysis between biophysical metrics of DP OT1 thymocyte interacting pMHC without or with CD8 cooperation and biological activity.**

**(A-AC)** Three combinations of plots were made: 1) CD8^-^ parameters *vs* CD8^-^ function (A-I), 2) CD8^-^ parameters *vs* CD8^+^ function (J-R), 3) CD8^+^ parameters *vs* CD8^+^ function (S-AC). Biophysical metrics of OT1 TCR on DP thymocytes binding to a panel of 5-7 peptides (OVA, Q4R7, T4, Q4H7, G4, in descending order of *x*-axis values) presented by H2-K^b^α3A2 or H2-K^b^ to prevent or permit CD8 bind MHC, are plotted *vs* the logarithm of the reciprocal peptide concentration required to stimulate half-maximal CD69 upregulation (1/EC50) and fitted by straight (red), exponential (green), and sigmoidal (blue) curves (different curves sometimes coincide, hence one obscuring the other). Biophysical metrics were measured by BFP force-clamp assay – optimal force $F_{\mathrm{opt}}$ (A, J, and S), catch bond intensity $I$ (B, K, and T), area under the curve $AUC$ (C, L, and U), peak bond lifetime $t_{\mathrm{peak}}$ (D, M, and V), and maximum slope $S_{\max}$ (E, N, and W); micropipette adhesion frequency assay – normalized average number of total bonds at zero force $\frac{\left\langle n \right\rangle_{\mathrm{tot}}}{\left( m_{\mathrm{TCR}}m_{\mathrm{pMHC}} \right)}$ (F, O, and X) and at $F_{\mathrm{opt}}$ $\frac{\left\langle n \right\rangle_{\mathrm{tot}}\left( F_{\mathrm{opt}} \right)}{\left( m_{\mathrm{TCR}}m_{\mathrm{pMHC}} \right)}$ (G, P, and Y), normalized synergy at zero force $\frac{\left\langle n \right\rangle_{\mathrm{tri}}}{\left( m_{\mathrm{TCR}}m_{\mathrm{pMHC}} \right)}$ (Z) and at $F_{\mathrm{opt}} \frac{\left\langle n \right\rangle_{\mathrm{tri}}\left( F_{\mathrm{opt}} \right)}{\left( m_{\mathrm{TCR}}m_{\mathrm{pMHC}} \right)}$ (AA); tetramer binding – 3D avidity $K_{V}$ (H, Q, and AB) and dwell time $t_{1/2}$ (I, R, and AC). *R*^2^ values for the three curve-fits, shown by matched colors, *P*-values indicating the statistically significant levels of fitting curves, and the Spearman’s rank correlation coefficient *ρ* are shown in each panel to gauge the level of correlation and goodness-of-fit. The CD69 upregulation and 3D binding parameters ($K_{V}$ and $t_{1/2}$) are from (9). The 2D binding parameters are either directly taken from (2) or calculated $\frac{\left\langle n \right\rangle_{\mathrm{tot}}}{\left( m_{\mathrm{TCR}}m_{\mathrm{pMHC}} \right)}$, $\frac{\left\langle n \right\rangle_{\mathrm{tri}}}{\left( m_{\mathrm{TCR}}m_{\mathrm{pMHC}} \right)}$, $F_{\mathrm{opt}}$, $I$, $AUC$, $t_{\mathrm{peak}}$, and $S_{\max}$ by fitting the data from (2) using the two-pathway model (cf. Supplementary Figure 3B). **(AD)** *1^st^ column*: List of 11 biophysical parameters measured by micropipette adhesion frequency, BFP force-clamp, and tetramer binding assays. *2^nd^ – 12^th^ columns*: two quantifiers ($x_{0}$ (midpoint of the sigmoidal fits) and fold-change of $1/k$ ($k$ = slope at $x_{0}$ of the sigmoidal fits)) for the three cases of CD8^+^ *vs* CD8^+^, CD8^-^ *vs* CD8^+^, and CD8^-^ *vs* CD8^-^, which represent three combinations of measurements made in the presence (+) and absence (-) of CD8: 1) CD8^+^ parameters *vs* CD8^+^ function, 2) CD8^-^ parameters *vs* CD8^+^ function, and 3) CD8^-^ parameters *vs* CD8^-^ function. All correlation analyses were performed using CD69 upregulation readouts for DP thymocytes ((A-AC) and Figure 3 (A)).


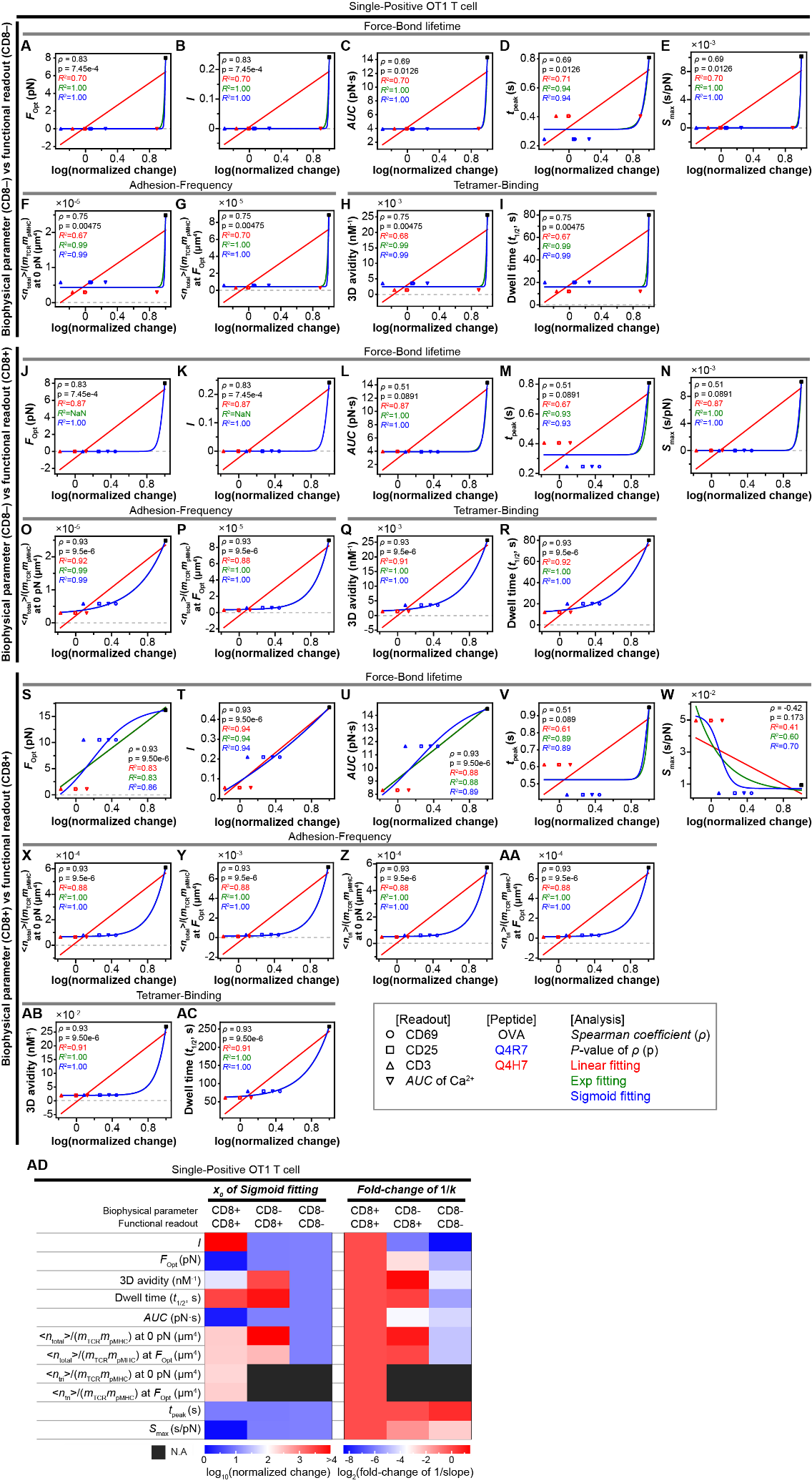


## Supplementary Figure 6. Correlating biophysical metrics of SP OT1 naïve T cells interaction with pMHC without or with CD8 cooperation and in the absence or presence of force with biological activity.

**(A-AC)** Three combinations of plots were made: 1) CD8^-^ parameters *vs* CD8^-^ function (A-I), 2) CD8^-^ parameters *vs* CD8^+^ function (J-R), 3) CD8^+^ parameters *vs* CD8^+^ function (S-AC). Biophysical metrics of OT1 TCR on SP naïve T cells binding to a panel of 3 peptides (OVA = black, Q4R7 = blue, Q4H7 = red) presented by H2-K^b^α3A2 or H2-K^b^ to prevent or permit CD8 bind MHC, are plotted *vs* the logarithm of normalized changes in expression of CD69 (circle), CD25 (square), CD3 (triangle), and intracellular calcium concentration (inverted triangle), and fitted by straight (red), exponential (green), and sigmoidal (blue) curves (different curves sometimes coincide, hence one obscuring the other). Biophysical metrics were measured by BFP force-clamp assay – optimal force $F_{\mathrm{opt}}$ (A, J, and S), catch bond intensity $I$ (B, K, and T), area under the curve $AUC$ (C, L, and U), peak bond lifetime $t_{\mathrm{peak}}$ (D, M, and V), and maximum slope $S_{\min}$ (E, N, and W); micropipette adhesion frequency assay – normalized average number of bonds at zero force $\frac{\left\langle n \right\rangle_{\mathrm{tot}}}{\left( m_{\mathrm{TCR}}m_{\mathrm{pMHC}} \right)}$ (F, O, and X) and at $F_{\mathrm{opt}} \frac{\left\langle n \right\rangle_{\mathrm{tot}}\left( F_{\mathrm{opt}} \right)}{\left( m_{\mathrm{TCR}}m_{\mathrm{pMHC}} \right)}$ (G, P, and Y), normalized synergy at zero force $\frac{\left\langle n \right\rangle_{\mathrm{tri}}}{\left( m_{\mathrm{TCR}}m_{\mathrm{pMHC}} \right)}$ (Z) and at $F_{\mathrm{opt}}$ $\frac{\left\langle n \right\rangle_{\mathrm{tri}}\left( F_{\mathrm{opt}} \right)}{\left( m_{\mathrm{TCR}}m_{\mathrm{pMHC}} \right)}$ (AA); tetramer binding – 3D avidity $K_{V}$ (H, Q, and AB) and dwell time $t_{1/2}$ (I, R, and AC). *R*^2^ values for the three curve-fits, shown by matched colors, *p*-values indicating the statistically significant levels of fitting curves, and the Spearman’s rank correlation coefficient *ρ* are shown in each panel to gauge the level of correlation and goodness-of-fit. The T cell functional responses are normalized from the data shown in Supplementary Figure 4. The 3D binding parameters ($K_{V}$ and $t_{1/2}$) are from (9). The five catch bond metrics $F_{\mathrm{opt}}$, $I$, $AUC$, $t_{\mathrm{peak}}$, and $S_{\max}$ are determined by first fitting the force-dependent lifetime data shown in Supplementary Figure 3C using the two-pathway model and then calculating values based on their definition shown in Figure 1B. **(AD)**  *1^st^ column*: List of 11 biophysical parameters measured by micropipette adhesion frequency, BFP force-clamp, and tetramer binding assays. *2^nd^ – 12^th^ columns*: two quantifiers ($x_{0}$ (midpoint of the sigmoidal fits) and fold-change of $1/k$ ($k$ = slope at $x_{0}$ of the sigmoidal fits)) for the three cases of CD8^+^ *vs* CD8^+^, CD8^-^ *vs* CD8^+^, and CD8^-^ *vs* CD8^-^, which represent three combinations of measurements made in the presence (+) and absence (-) of CD8: 1) CD8^+^ parameters *vs* CD8^+^ function, 2) CD8^-^ parameters *vs* CD8^+^ function, and 3) CD8^-^ parameters *vs* CD8^-^ function. The correlation analyses were performed using functional readout(s): CD3, CD25, CD69, and Ca^2+^ $AUC$ for the SP naïve T cells ((A-AC) and Figure 3 (B)).


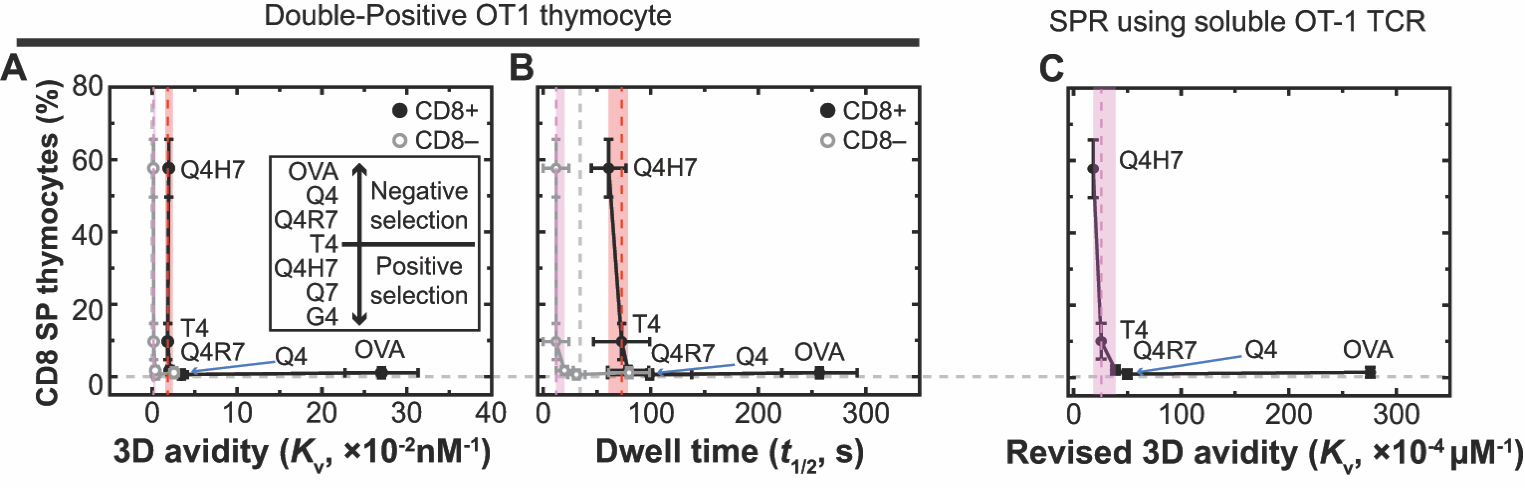


##

## Supplementary Figure 7. CD8 impacts TCR specificity for two ligands evaluated by 3D measures.

**(A, B, C)** The percentages of CD8^+^ SP thymocytes from FTOC assay are plotted *vs* 3D avidity $K_{V}$ (A), dwell time $t_{1/2}$ (B), or newly published 3D avidity $K_{V}$ values (C) from (10) of OT1 TCR interacting with the same panel of 7 peptides presented by H2-K^b^ (CD8^+^, closed black circles connected by black line segments) or H2-K^b^α3A2 (CD8^-^, open gray circles connected by gray line segments) to permit or prevent CD8 from binding to MHC. Vertical dashed lines identify the parameters of the threshold peptide (T4) and the vertical stripes mark the parameter ranges across the threshold from the strongest positive selection peptide (Q4H7) to the weakest negative selection peptide (Q4R7) using different colors to indicate measurements made when CD8 was prevented (purple) or permitted (red) to bind MHC. All data of 3D avidity, dwell time, and FTOC assays are from the literature (9).


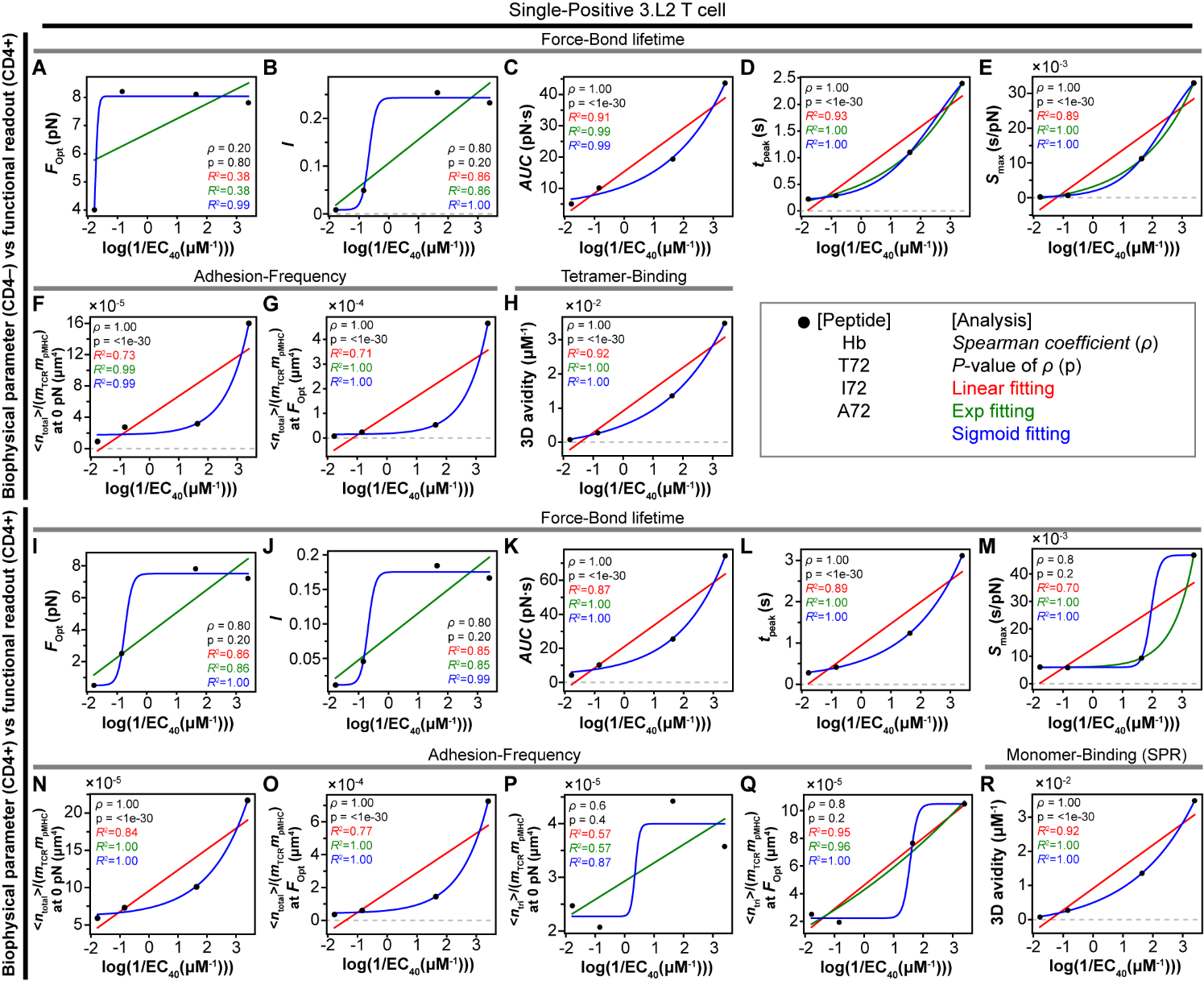

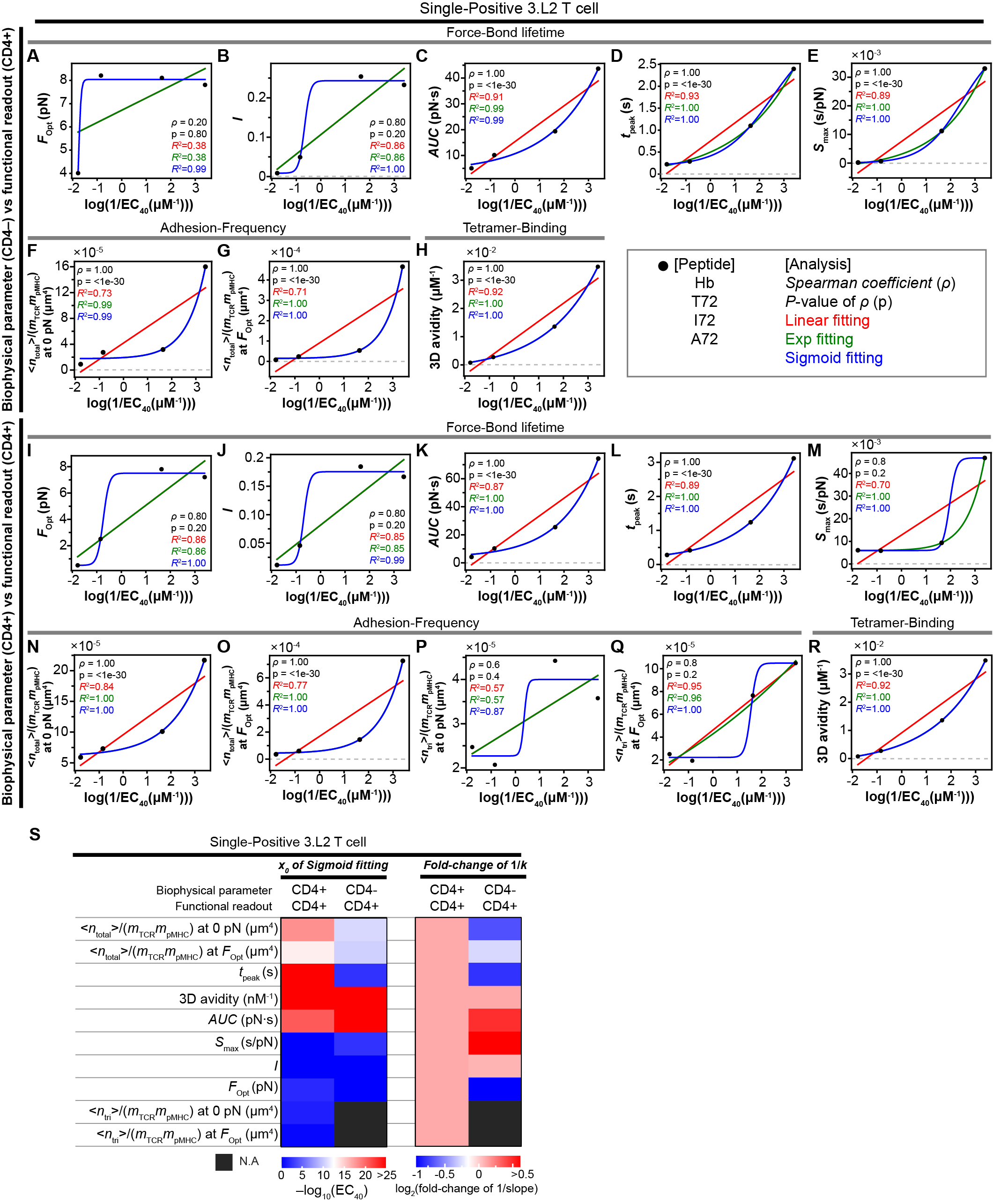


## Supplementary Figure 8. Correlating biophysical metrics of 3.L2 TCR T cells interaction with pMHC without or with CD4 cooperation and in the absence or presence of force with biological activity.

**(A-Q)** Biophysical metrics measured using T cells from 3.L2 TCR transgenic mice respectively expressing CD4^-^CD8^+^ to prevent (CD4-, A-H) or CD4^+^CD8^-^ to permit (CD4+, I-R) CD4 to bind I-E^k^ that presents WT peptide Hb_64-78_ and its MTs T72, I72, and A72, by micropipette adhesion frequency assay (F, G, N, O, P, Q) and BFP force-clamp assay (A-E and I-M) – effective 2D affinity at zero-force *A*_c_*K*_a_ (F, N), effective 2D affinity at optimal force *A*_c_*K*_a_($F_{\mathrm{opt}}$) = *A*_c_*K*_a_ $\times k_{\mathrm{off}}\times t_{\mathrm{peak}}$ (G, O), normalized synergy at zero force $\frac{\left\langle n \right\rangle_{\mathrm{tri}}}{\left( m_{\mathrm{TCR}}m_{\mathrm{pMHC}} \right)}$ (P) and at $F_{\mathrm{opt}}$ $\frac{\left\langle n \right\rangle_{\mathrm{tri}}\left( \mathrm{at}F_{\mathrm{opt}} \right)}{\left( m_{\mathrm{TCR}}m_{\mathrm{pMHC}} \right)}$ (Q), optimal force $F_{\mathrm{opt}}$ (A, I), catch bond intensity *I* (B, J), area under the curve *AUC* (C, K), peak bond lifetime $t_{\mathrm{peak}}$ (D, L), and maximum slope $S_{\max}$ (E, M) – are plotted vs the logarithm of the reciprocal peptide concentration required to stimulate 40% maximal IL2 (1/EC40) and fitted by straight (red), exponential (green) and sigmoidal (blue) curves (different curves sometimes coincide, hence one obscuring the other). *R*^2^ values for the three curve-fits, shown by matched colors, *P*-values indicating the statistically significant levels of fitting curves, and the Spearman’s rank correlation coefficient *ρ* are shown in each panel to gauge the level of correlation and goodness-of-fit. The data in the plots were from re-analysis of the original data published in (1, 7). **(R)** The same correlative analysis was performed using the 3D avidity as the ***y***-axis variable from (8). **(S)** *1^st^ column*: List of 10 biophysical parameters measured by micropipette adhesion frequency, BFP force-clamp, and surface plasmon resonance (SPR). *2^nd^ – 9^th^ columns*: two quantifiers ($\boldsymbol{x}_{\boldsymbol{0}}$ (midpoint of the sigmoidal fits) and fold-change of $\boldsymbol{1/k}$ ($\boldsymbol{k}$ = slope at $\boldsymbol{x}_{\boldsymbol{0}}$ of the sigmoidal fits)) for the two cases of CD4^+^ *vs* CD4^+^ and CD4^-^ *vs* CD4^+^. The correlation analyses were performed using the logarithm of the reciprocal peptide concentration required to stimulate 40% maximal IL2 (1/EC40) as functional readout(s) ((A-R) and Figure 6). The functional readout is directly from (11).


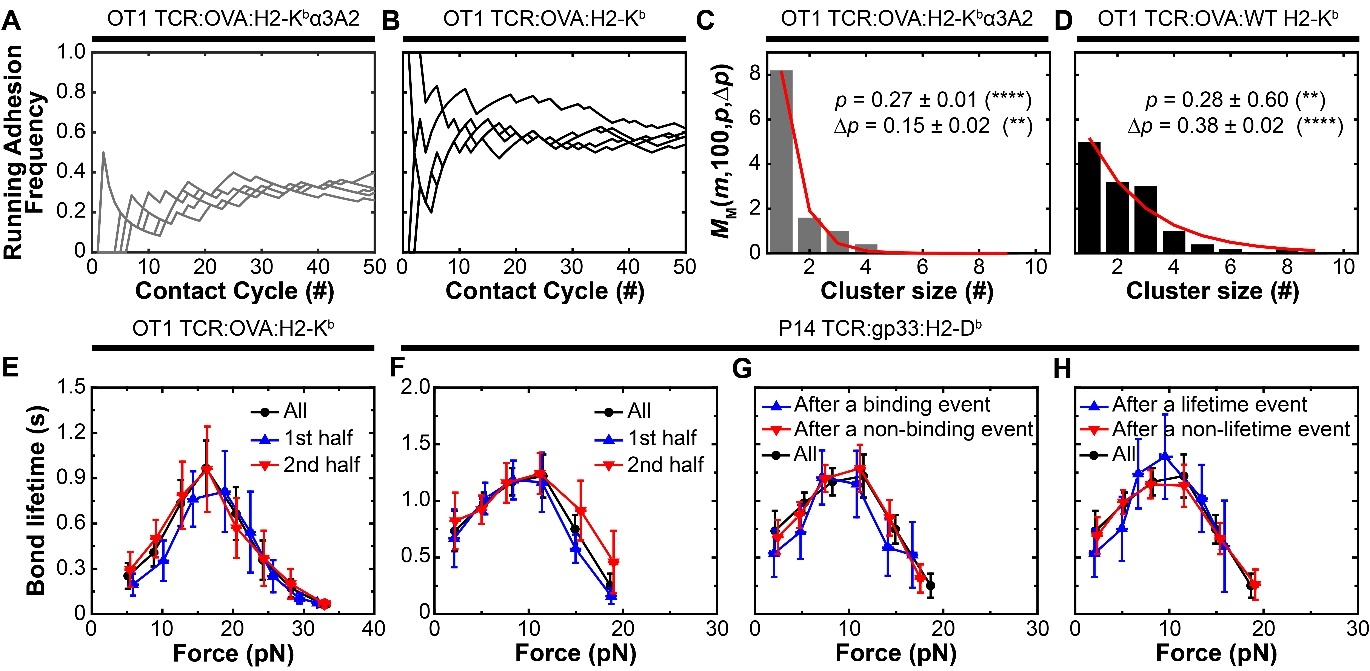


## Supplementary Figure 9. Analysis of molecular memory in the adhesion frequency and force-dependent bond lifetimes.

**(A, B)** Representative running adhesion frequency *vs* cycle number of repeated contacts between individual naïve OT1 T cells and red blood cells coated with OVA peptide presented by MT H2-K^b^α3A2 (A) or WT H2-K^b^ (B). (**C, D**) Adhesion cluster size distributions (bar) and model fit (curve) calculated from the above running adhesion frequency data obtained in the absence (C) and presence (D) of CD8 contribution using the method described in (12, 13). ***P*<0.01, *****P*<0.0001 indicating the statistically significant levels of fitting curves. **(E, F)** Mean ± SEM of single bond lifetimes of OT1 naïve T cells interacting with OVA_257-264_:H2-K^b^ (E) or P14 naïve T cells interacting with gp33^41M^:H2-D^b^ (F) measured from the first half (blue triangle), last half (red inverted triangle), and all (black circle) lifetime events in the time series generated by repetitively testing a single cell. **(G, H)** Mean ± SEM of lifetimes of single P14 TCR–gp33^41M^:H2-D^b^ bonds measured after a binding (G) or lifetime (H) event (blue triangle), or after a non-binding (G) or non-lifetime (H) event (red inverted triangle), and all events (black circle). Data are pooled from 10 (E) and 30 (F-H) cells with 5-25 measurements per cell.


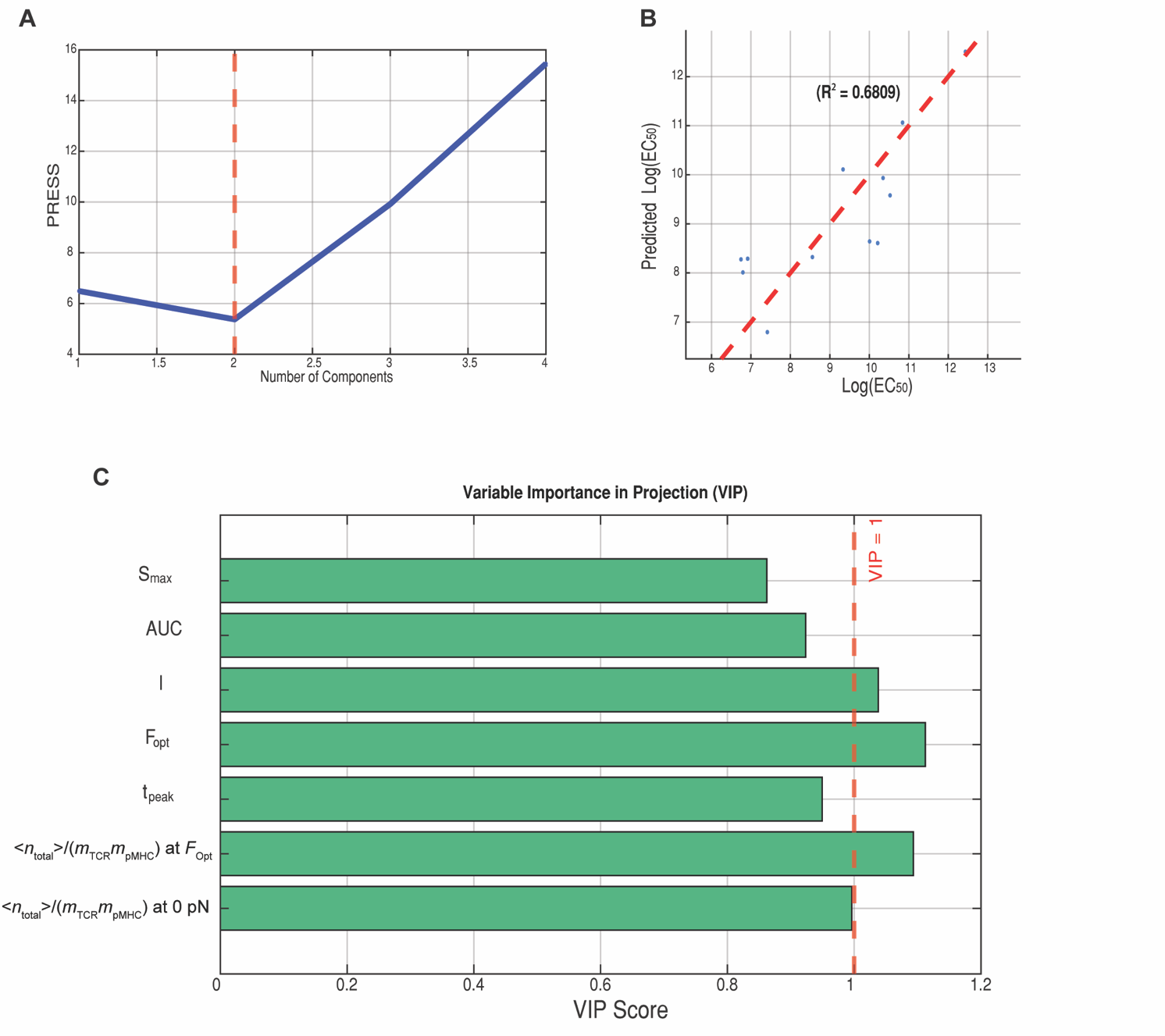


**Supplementary Figure 10. Partial Least Squares (PLS) regression identifies optimal linear combinations of force-based metrics as primary predictors of T cell activation.**

**(A)** Leave-One-Out Cross-Validation (LOOCV) of the PLS model using the combined CD8+ and CD8- double-positive thymocyte dataset (n = 12). The Predicted Residual Error Sum of Squares (PRESS) minimizes at precisely two latent components (red dashed line), identifying the optimal model complexity required to capture underlying biophysical relationships without overparameterization. **(B)** Predictive performance of the final 2-component PLS model. Predicted versus actual values for the T cell activation readout (log_10_(EC_50_ of CD69)) demonstrate a robust fit (R² = 0.6809). All seven biophysical input parameters were mean-centered and variance-scaled prior to model construction to ensure unbiased weighting. **(C)** Variable Importance in Projection (VIP) scores evaluating the true contribution of each biophysical parameter to the predictive model. The VIP score aggregates the weight coefficients across the latent components, with scores > 1 (red dashed line) denoting highly influential variables.

| TCR | peptide | MHC | Cell type | Measurement Techniques | Figures |
| --- | --- | --- | --- | --- | --- |
| OT1 | OVA | H2-Kb | CD4+CD8+ (double positive, DP) thymocytes | Micropipette adhesion frequency and BFP force-clamp (2)  CD69 upregulation, FTOC, and tetramer binding (9)  SPR (10) | 1-5, S3, S5, S7, and S10. |
| OT1 | Q4 | H2-Kb |  |  |  |
| OT1 | Q4R7 | H2-Kb |  |  |  |
| OT1 | T4 | H2-Kb |  |  |  |
| OT1 | Q4H7 | H2-Kb |  |  |  |
| OT1 | Q7 | H2-Kb |  |  |  |
| OT1 | G4 | H2-Kb |  |  |  |
| OT1 | OVA | H2-Kb | CD8+ (single positive, SP) naïve T cells | Micropipette adhesion frequency (8)  BFP force-clamp (this study and (3)  TGT Calcium, CD69/25 upregulation CD3 downregulation (this study)  Tetramer binding (9) | 2-3, S1, S3-S4, S6, and S9. |
| OT1 | Q4R7 | H2-Kb |  |  |  |
| OT1 | Q4H7 | H2-Kb |  |  |  |
| 2C | R4 | H2-Kb | CD4+CD8+ (double positive, DP) thymocytes | BFP force-clamp (2) | 2 and S3. |
| 2C | dEV8 | H2-Kb |  |  |  |
| 2C | EVSV | H2-Kb |  |  |  |
| P14 | gp33 (41M) | H-2Db | CD8+ naïve T cells | BFP force-clamp (4) | 2 and S3. |
| P14 | gp33 (41C) | H-2Db |  |  |  |
| P14 | gp33 (41CGI) | H-2Db |  |  |  |
| N15 | VSV8 | H2-Kb | CD8+ T cells | Optical Tweezers (6) | 2 and S3. |
| B13.C1 | NP366 | H-2Db | Hybridomas | BFP force-clamp (5) | 2 and S3. |
| B17.C1 | NP366 | H-2Db |  |  |  |
| B17.R1^†^ | NP366 | H-2Db |  |  |  |
| B17.R2^†^ | NP366 | H-2Db |  |  |  |
| 3.L2 | Hb | I-Ek | CD4-CD8+ or CD4+CD8- naïve T cells | Micropipette adhesion frequency, BFP force-clamp, and SPR (7, 8)  Peptide dependent B cell apoptosis (11) | 6, S3, and S8. |
| 3.L2 | T72 | I-Ek |  |  |  |
| 3.L2 | I72 | I-Ek |  |  |  |
| 3.L2 | A72 | I-Ek |  |  |  |
| E8 | TPI | HLA-DR1 | Cell-free system and Jurkat T cells | BFP force-clamp (1) | 6 and S2. |

## Supplementary Table 1. Summary of all TCR:pMHC pairs analyzed in this study.

| **Super agonist ~ Agonist** | | | **Weak agonist** | | | **Antagonist** | | |
| --- | --- | --- | --- | --- | --- | --- | --- | --- |
| TCR | peptide | MHC Ⅰ* | TCR | peptide | MHC Ⅰ* | TCR | peptide | MHC Ⅰ* |
| N15 | VSV8 | H-2K^b^ | OT1 | Q4 | H-2K^b^ | OT1 | G4 | H-2K^b^ |
| P14 | gp33^41M^ | H-2D^b^ | OT1 | Q4R7 | H-2K^b^ | 2C | EVSV | H-2K^b^ |
| P14 | gp33^41C^ | H-2D^b^ | OT1 | T4 | H-2K^b^ | P14 | gp33^41CGI^ | H-2D^b^ |
| OT1 | OVA | H-2K^b^ | OT1 | Q4H7 | H-2K^b^ | B17.R1^†^ | NP366 | H-2D^b^ |
| 2C | R4 | H-2K^b^ | OT1 | Q7 | H-2K^b^ | B17.R2^†^ | NP366 | H-2D^b^ |
| 2C | Dev8 | H-2K^b^ | P14 | gp33^41C^ | H-2D^b^ |  |  |  |
| B13.C1 | NP366 | H-2D^b^ |  |  |  |  |  |  |
| B17.C1 | NP366 | H-2D^b^ |  |  |  |  |  |  |

## Supplementary Table 2. Summary of TCRs and pMHC-Is and their classification.

*****MHCs include WT and mutant (α3A2) to abolish CD8 binding.

†Reverse-docking topology of TCRs are considered as antagonist albeit the peptide (NP366) is known as agonist for canonical-docking topology of TCR binding to MHC.

# References

1. Rushdi MN, Pan V, Li K, Choi H-K, Travaglino S, Hong J, et al. Cooperative binding of T cell receptor and CD4 to peptide-MHC enhances antigen sensitivity. Nature communications. 2022;13(1):7055.

2. Hong J, Ge C, Jothikumar P, Yuan Z, Liu B, Bai K, et al. A TCR mechanotransduction signaling loop induces negative selection in the thymus. Nature immunology. 2018;19(12):1379-90.

3. Liu B, Chen W, Evavold Brian D, Zhu C. Accumulation of Dynamic Catch Bonds between TCR and Agonist Peptide-MHC Triggers T Cell Signaling. Cell. 2014;157(2):357-68.

4. Kolawole EM, Andargachew R, Liu B, Jacobs JR, Evavold BD. 2D kinetic analysis of TCR and CD8 coreceptor for LCMV GP33 epitopes. Frontiers in Immunology. 2018;9:408914.

5. Zareie P, Szeto C, Farenc C, Gunasinghe SD, Kolawole EM, Nguyen A, et al. Canonical T cell receptor docking on peptide–MHC is essential for T cell signaling. Science. 2021;372(6546):eabe9124.

6. Das DK, Feng Y, Mallis RJ, Li X, Keskin DB, Hussey RE, et al. Force-dependent transition in the T-cell receptor β-subunit allosterically regulates peptide discrimination and pMHC bond lifetime. Proceedings of the National Academy of Sciences. 2015;112(5):1517-22.

7. Hong J, Persaud SP, Horvath S, Allen PM, Evavold BD, Zhu C. Force-regulated in situ TCR–peptide-bound MHC class II kinetics determine functions of CD4+ T cells. The Journal of Immunology. 2015;195(8):3557-64.

8. Hong JS. 2D kinetics and force regulation study of T cell recognition and thymocyte selection: Georgia Institute of Technology; http://hdl.handle.net/1853/53430; 2014.

9. Daniels MA, Teixeiro E, Gill J, Hausmann B, Roubaty D, Holmberg K, et al. Thymic selection threshold defined by compartmentalization of Ras/MAPK signalling. Nature. 2006;444(7120):724-9.

10. Huhn A, Kutuzov MA, Maclean K, Uhl LF, Mahale JM, Gérard A, et al. Murine T-cell receptor OT-I exhibits imperfect discrimination between foreign and self-antigens. The EMBO Journal. 2025;45(2):394.

11. Kersh GJ, Allen PM. Structural basis for T cell recognition of altered peptide ligands: a single T cell receptor can productively recognize a large continuum of related ligands. The Journal of experimental medicine. 1996;184(4):1259-68.

12. Zarnitsyna VI, Huang J, Zhang F, Chien Y-H, Leckband D, Zhu C. Memory in receptor–ligand-mediated cell adhesion. Proceedings of the National Academy of Sciences. 2007;104(46):18037-42.

13. Rosado AM, Zhang Y, Choi H-K, Chen Y, Ehrlich SM, Jin F, et al. Memory in repetitive protein–protein interaction series. APL Bioengineering. 2023;7(1).
